# Supplementary material for: Snakebites in “Invisible Populations”: A cross-sectional survey in riverine populations in the remote western Brazilian Amazon
Source: PLoS Negl Trop Dis. 2021 Sep 9;15(9):e0009758. doi: 10.1371/journal.pntd.0009758 (PMC8454940; doi:10.1371/journal.pntd.0009758)
Supplement: S1 File — (DOC) [file pntd.0009758.s001.doc]

STROBE Statement—Checklist of items that should be included in reports of ***cross-sectional studies***

|  | Item No | Recommendation |
| --- | --- | --- |
| **Title and abstract** | 1 | (*a*) Indicate the study’s design with a commonly used term in the title or the abstract - **study’s design was indicated in the title** |
|  |  | (*b*) Provide in the abstract an informative and balanced summary of what was done and what was found **– an abstract was presented in the first page** |
| Introduction | | |
| Background/rationale | 2 | Explain the scientific background and rationale for the investigation being reported **– an Introduction section was presented after the abstract** |
| Objectives | 3 | State specific objectives, including any prespecified hypotheses **– study objectives were presented at the end of the Introduction section** |
| Methods | | |
| Study design | 4 | Present key elements of study design early in the paper – **A ‘Study design and sampling’ section is presented at Methods** |
| Setting | 5 | Describe the setting, locations, and relevant dates, including periods of recruitment, exposure, follow-up, and data collection **A ‘Study sites, access to communities and subjects’ section is presented at Methods** |
| Participants | 6 | (*a*) Give the eligibility criteria, and the sources and methods of selection of participants - **A ‘Study design and sampling’ section is presented at Methods** |
| Variables | 7 | Clearly define all outcomes, exposures, predictors, potential confounders, and effect modifiers. Give diagnostic criteria, if applicable – **A ‘Data collection’ section is presented at Methods** |
| Data sources/ measurement | 8* | For each variable of interest, give sources of data and details of methods of assessment (measurement). Describe comparability of assessment methods if there is more than one group - **A ‘Data collection’ section is presented at Methods** |
| Bias | 9 | Describe any efforts to address potential sources of bias - **A ‘Data collection’ section is presented at Methods** |
| Study size | 10 | Explain how the study size was arrived at - **A ‘Study design and sampling’ section is presented at Methods** |
| Quantitative variables | 11 | Explain how quantitative variables were handled in the analyses. If applicable, describe which groupings were chosen and why - **A ‘Data analysis’ section is presented at Methods** |
| Statistical methods | 12 | (*a*) Describe all statistical methods, including those used to control for confounding - **A ‘Data analysis’ section is presented at Methods** |
|  |  | (*b*) Describe any methods used to examine subgroups and interactions - **A ‘Data analysis’ section is presented at Methods** |
|  |  | (*c*) Explain how missing data were addressed - **A ‘Data analysis’ section is presented at Methods** |
|  |  | (*d*) If applicable, describe analytical methods taking account of sampling strategy - **A ‘Data analysis’ section is presented at Methods** |
|  |  | (*e*) Describe any sensitivity analyses - **A ‘Data analysis’ section is presented at Methods** |
| Results | | |
| Participants | 13* | (a) Report numbers of individuals at each stage of study—eg numbers potentially eligible, examined for eligibility, confirmed eligible, included in the study, completing follow-up, and analysed - **A ‘Characteristics of the participants’ section is presented at Results** |
|  |  | (b) Give reasons for non-participation at each stage - **A ‘Characteristics of the participants’ section is presented at Results** |
|  |  | (c) Consider use of a flow diagram – **Not used because of the few steps in the inclusion process; a ‘Characteristics of the participants’ section is presented at Results** |
| Descriptive data | 14* | (a) Give characteristics of study participants (eg demographic, clinical, social) and information on exposures and potential confounders - **A ‘Characteristics of the participants’ section is presented at Results** |
|  |  | (b) Indicate number of participants with missing data for each variable of interest - **A ‘Characteristics of the participants’ section is presented at Results** |
| Outcome data | 15* | Report numbers of outcome events or summary measures – **Sections of outcomes (‘Access to healthcare and associated factors’ and ‘Deaths from snakebites’) are present at Results** |
| Main results | 16 | (*a*) Give unadjusted estimates and, if applicable, confounder-adjusted estimates and their precision (eg, 95% confidence interval). Make clear which confounders were adjusted for and why they were included – **Section of ‘Access to healthcare and associated factors’ is present at Results** |
|  |  | (*b*) Report category boundaries when continuous variables were categorized **– Not applicable** |
|  |  | (*c*) If relevant, consider translating estimates of relative risk into absolute risk for a meaningful time period **– Not applicable** |
| Other analyses | 17 | Report other analyses done—eg analyses of subgroups and interactions, and sensitivity analyses **– Not applicable** |
| Discussion | | |
| Key results | 18 | Summarise key results with reference to study objectives **– See section of ‘Invisible populations, hidden burden of snakebites’ at Discussion** |
| Limitations | 19 | Discuss limitations of the study, taking into account sources of potential bias or imprecision. Discuss both direction and magnitude of any potential bias – **Section of ‘Limitations’ is present at Discussion** |
| Interpretation | 20 | Give a cautious overall interpretation of results considering objectives, limitations, multiplicity of analyses, results from similar studies, and other relevant evidence **– See Discussion section at all** |
| Generalisability | 21 | Discuss the generalisability (external validity) of the study results - **See Discussion section at all (especially paragraphs 5, 7, 8 and 9 of this section)** |
| Other information | | |
| Funding | 22 | Give the source of funding and the role of the funders for the present study and, if applicable, for the original study on which the present article is based - **A ‘Funding’ section is presented after ‘Concluding remarks’** |
