# Supplementary material for: Snakebites in “Invisible Populations”: A cross-sectional survey in riverine populations in the remote western Brazilian Amazon
Source: PLoS Negl Trop Dis. 2021 Sep 9;15(9):e0009758. doi: 10.1371/journal.pntd.0009758 (PMC8454940; doi:10.1371/journal.pntd.0009758)
Supplement: S3 File — (DOCX) [file pntd.0009758.s003.docx]

**Supporting Information 3.** Traditional medicines used by the study participants.

| **Traditional medicine, administration** | **Solimões River** | | **Juruá River** | | **Purus River** | |
| --- | --- | --- | --- | --- | --- | --- |
|  | Number | % | Number | % | Number | % |
| *Específico Pessoa*, oral | 10 | 25.0 | 2 | 3.3 | 2 | 17.0 |
| *Específico Pessoa*, oral + Orange (*Citrus aurantium*) peel infusion, oral | 3 | 7.5 | … | … | … | … |
| *Específico Pessoa*, oral + Needlegrass, called ‘barba de bode’ in Portuguese (*Aristida longiseta*) infusion, oral | 2 | 5.0 | … | … | … | … |
| ‘Panacari’ in Portuguese (*Peltodon radicans*) leaves infusion, oral | 2 | 5.0 | … | … | … | … |
| *Específico Pessoa*, oral + Benzylpenicillin, parenteral | 2 | 5.0 | … | … | … | … |
| Cow milk, oral | 1 | 2.5 | … | … | 1 | 8.3 |
| Gall bladder of agouti (*Cuniculus paca*), oral + Lemon grass (*Cymbopogon flexuosus*) leaves infusion + Tambaqui (*Colossoma macropomum*) lard, topical + Tambaqui (*Colossoma macropomum*) skin, infusion | 1 | 2.5 | … | … | … | … |
| Peach tomato, called ‘cubiu’ in Portuguese (*Solanum sessiliflorum*), topical | 1 | 2.5 | … | … | … | … |
| Metamizole, oral + Salt water, oral | 1 | 2.5 | … | … | … | … |
| Específico Pessoa, oral + ‘Pau de colher’ in Portuguese (*Lacmellea arborescens*) leaves infusion, oral + Mulungu (*Erythrina mulungu*) bark and leaves, infusion, oral | 1 | 2.5 | … | … | … | … |
| Blessings from a faith healer + Water with lemon | 1 | 2.5 | … | … | … | … |
| Black cane (?) sap + Açaí tree's eye (young central foliage of the plant) (*Euterpe oleracea*) sap, oral | 1 | 2.5 | … | … | … | … |
| Açaí tree's root (*Euterpe oleracea*) + ‘crajiru’ or ‘pariri’ in Portuguese (Arrabidaea chica) leaves, infusion, oral | 1 | 2.5 | … | … | … | … |
| Bellyache bush, called ‘pião’ or ‘pinhão’ in Portuguese (*Jatropha gossipifolia*) leaves infusion, oral | 1 | 2.5 | … | … | … | … |
| Peach tomato, called ‘cubiu’ in Portuguese (*Solanum sessiliflorum*) infusion, oral + Salt | 1 | 2.5 | … | … | … | … |
| Açaí tree's eye (young central foliage of the plant) (*Euterpe oleracea*) sap, oral | 1 | 2.5 | … | … | … | … |
| Aloe (*Aloe vera*), called ‘babosa’ in Portuguese, bark of the juicy leaves infusion, oral | 1 | 2.5 | … | … | … | … |
| Gall bladder of agouti (*Cuniculus paca*), oral + Lemon grass (*Cymbopogon flexuosus*) leaves infusion | 1 | 2.5 | … | … | … | … |
| Armoured catfish (‘bodó’ in Portuguese) (Siluriformes, Loricariidae), fish lard, infusion | 1 | 2.5 | … | … | … | … |
| *Específico Pessoa* + prayers | 1 | 2.5 | … | … | … | … |
| Benzylpenicillin, parenteral | 1 | 2.5 | … | … | … | … |
| Cow milk, oral + ‘Moon light power’ | 1 | 2.5 | … | … | … | … |
| ‘Mari’ seed ([*Poraqueiba sericea*](https://pt.wikipedia.org/wiki/Poraqueiba_sericea)), infusion, oral | 1 | 2.5 | … | … | … | … |
| Caiman leather (Crocodylia, Alligatoridae), infusion, oral | 1 | 2.5 | … | … | … | … |
| Snake guts, topical + Snake skin strapped to the leg + Snake skin ingestion | 1 | 2.5 | … | … | … | … |
| Black stone (or ‘snake stone’), topical | 1 | 2.5 | 7 | 11.7 | … | … |
| Chicory root (*Cichorium intybus*) infusion, oral | … | … | 3 | 5.0 | … | … |
| Alligator apple, called ‘araticum’ in Portuguese (*Annona* sp.) bark and leaves, infusion, oral + Scraped material from the surfaces of the second step of a wooden staircase, which gives access from the river to the community, infusion, oral | … | … | 3 | 5.0 | … | … |
| *Específico Pessoa* + Scraped material from the surfaces of the second step of a wooden staircase, which gives access from the river to the community, infusion, oral | … | … | 3 | 5.0 | … | … |
| Açaí tree's young fruit (*Euterpe oleracea*) infusion, oral | … | … | 2 | 3.3 | … | … |
| *Específico Pessoa* + Chicory root (*Cichorium intybus*) infusion, oral + Scraped material from the surfaces of the second step of a wooden staircase, which gives access from the river to the community, infusion, oral + Açaí tree's fruit (*Euterpe oleracea*) infusion, oral | … | … | 2 | 3.3 | … | … |
| Chicory root (*Cichorium intybus*) infusion, oral + ‘Mulambinho’ (*Polyscias fruticosa?)* root, infusion, oral+ Açaí tree (*Euterpe oleracea*) sap, oral | … | … | 2 | 3.3 | … | … |
| *Específico Pessoa* + Açaí tree's fruit (*Euterpe oleracea*) infusion, oral | … |  | 2 | 3.3 |  |  |
| *Específico* 33 + Açaí tree (*Euterpe oleracea*) sap, oral | … | … | 2 | 3.3 | … | … |
| Spiked spiralflag ginger, called ‘canarana’ in Portuguese, rhizome infusion, oral | … | … | 2 | 3.3 | … | … |
| *Específico Pessoa* + Açaí tree's fruit (*Euterpe oleracea*) infusion, oral + Scraped material from the surfaces of the second step of a wooden staircase, which gives access from the river to the community, infusion, oral | … | … | 2 | 3.3 | … | … |
| Snake guts, topical | … | … | 1 | 1.7 | … | … |
| Açaí tree's eye (young central foliage of the plant) (*Euterpe oleracea*) infusion, oral | … | … | 1 | 1.7 | … | … |
| Footed tortoise broth, called ‘jabuti’ in Portuguese (*Chelonoidis* spp.), oral | … | … | 1 | 1.7 | … | … |
| Soursop (*Annona muricata*) leaves infusion, oral | … | … | 1 | 1.7 | … | … |
| Soursop (*Annona muricata*) leaves + ‘Envira’ barks (*Bocageopsis multiflora*) in Portuguese, infusion, oral | … | … | 1 | 1.7 | … | … |
| Raw coffee (*Coffea arabica*) bean infusion, topical + Benzylpenicillin, parenteral | … | … | 1 | 1.7 | … | … |
| Hot boiled egg, topical + Diclofenac, oral | … | … | 1 | 1.7 | … | … |
| *Específico Pessoa* + Ash containing embers in water, oral | … | … | 1 | 1.7 | … | … |
| Culicid mosquitos, called ‘carapanã’ in Portuguese, infusion, oral + Footed tortoise broth, called ‘jabuti’ in Portuguese (*Chelonoidis* spp.), oral + Scraped material from the surfaces of the second step of a wooden staircase, which gives access from the river to the community, infusion, oral | … | … | 1 | 1.7 | … | … |
| Alligator apple, called ‘araticum’ in Portuguese (*Annona* sp.) bark and leaves, infusion, oral + Açaí tree's root (*Euterpe oleracea*), infusion, oral + Diclofenac, oral + Metamizole, oral + Dexamethasone, oral | … | … | 1 | 1.7 | … | … |
| Culicid mosquitos, called ‘carapanã’ in Portuguese, infusion, oral + Footed tortoise broth, called ‘jabuti’ in Portuguese (*Chelonoidis* spp.), oral | … | … | 1 | 1.7 | … | … |
| Açaí tree (*Euterpe oleracea*) sap, oral + Tablets of Paracetamol + Acetylsalicylic Acid + Caffeine, oral | … | … | 1 | 1.7 | … | … |
| Chicory root (*Cichorium intybus*) infusion, oral + Footed tortoise broth, called ‘jabuti’ in Portuguese (*Chelonoidis* spp.), oral | … | … | 1 | 1.7 | … | … |
| Açaí tree's eye (young central foliage of the plant) (*Euterpe oleracea*) sap, oral + ‘Japana’ (*Ayapana triplinervis*) stems and leaves, infusion, oral | … | … | 1 | 1.7 | … | … |
| ‘Jucá’ (*Libididia ferrea*) leaves, infusion, oral + Mango tree (*Mangifera indica*) barks, infusion, oral + Candiru or vampire fish (*Vandellia cirrhosa*), dried fish, infusion, oral | … | … | 1 | 1.7 | … | … |
| *Específico Pessoa* + Ecuadorian ivory palm tree's eye (young central foliage of the plant) (*Phytelephas macrocarpa*) infusion, oral + Açaí tree's young fruits (*Euterpe oleracea*) infusion, oral | … | … | 1 | 1.7 | … | … |
| Açaí tree fruits (*Euterpe oleracea*), infusion, oral + Scraped material from the surfaces of the second step of a wooden staircase, which gives access from the river to the community, infusion, oral | … | … | 1 | 1.7 | … | … |
| Chicory root (*Cichorium intybus*) infusion, oral + ‘Moon light power’ | … | … | 1 | 1.7 | … | … |
| Chicory root (*Cichorium intybus*) infusion, oral + Black stone (or ‘snake stone’), topical + Antinflammatory (not remembered) | … | … | 1 | 1.7 | … | … |
| ‘Japana’ (*Ayapana triplinervis*) stems and leaves, infusion, oral + Benzylpenicillin, parenteral | … | … | 1 | 1.7 | … | … |
| Chicory root (*Cichorium intybus*) infusion, oral + Alligator apple, called ‘araticum’ in Portuguese (*Annona* sp.) bark and leaves, infusion, oral | … | … | 1 | 1.7 | … | … |
| Scraped material from the surfaces of the second step of a wooden staircase, which gives access from the river to the community, infusion, oral + ‘japana’ (*Ayapana triplinervis*) stems and leaves, infusion, oral | … | … | 1 | 1.7 | … | … |
| Chicory root (*Cichorium intybus*) infusion, oral + Scraped material from the surfaces of the second step of a wooden staircase, which gives access from the river to the community, infusion, oral + Bushy matgrass (‘erva cidreira’ or ‘carmelitana’, in Portuguese) (*Lippia alba*) leaves, infusion, oral | … | … | 1 | 1.7 | … | … |
| Gunpowder tea, oral + Scraped material from the surfaces of the second step of a wooden staircase, which gives access from the river to the community, infusion, oral | … | … | 1 | 1.7 | … | … |
| ‘Japana’ (*Ayapana triplinervis*) stems and leaves, infusion, oral | … | … | 1 | 1.7 | … | … |
| Spiked spiralflag ginger, called ‘canarana’ in Portuguese, rhizome infusion, oral + Açaí tree fruits (*Euterpe oleracea*), infusion, oral | … | … | 1 | 1.7 | … | … |
| Açaí tree's eye (young central foliage of the plant) (*Euterpe oleracea*) sap, oral + Salt water, oral | … | … | 1 | 1.7 | … | … |
| Footed tortoise, called ‘jabuti’ in Portuguese (*Chelonoidis* spp.), shell, infusion, oral + Footed tortoise, called ‘jabuti’ in Portuguese (*Chelonoidis* spp.), meat, oral | … | … | 1 | 1.7 | … | … |
| Indian bark (?) infusion, oral + Pirarucu (*Arapaima gigas*) lard, topical | … | … | … | … | 1 | 8.3 |
| Cururu frog (*Rhinella marina*) leather infusion, oral + Homemade hydration solution (‘soro caseiro’, in Portuguese), oral | … | … | … | … | 1 | 8.3 |
| Gasoline, topical | … | … | … | … | 1 | 8.3 |
| ‘Cipó-de-tracuá’ in Portuguese (*Philodendron megalophyllum*), stem infusion, oral | … | … | … | … | 1 | 8.3 |
| Bellyache bush, called ‘pião’ or ‘pinhão’in Portuguese (*Jatropha gossipifolia*) leaves sap, oral | … | … | … | … | 1 | 8.3 |
| Tambaqui (*Colossoma macropomum*) scales, infusion, oral + Yellow-spotted river turtle, called ‘tracajá’ in Portuguese (*Podocnemis unifilis*), bonés, infusion, oral | … | … | … | … | 1 | 8.3 |
| Tourniquet + ‘Moon light power’ | … | … | … | … | 1 | 8.3 |
| *Específico Pessoa* + Cow milk, oral | … | … | … | … | 1 | 8.3 |
| Pirarucu (*Arapaima gigas*) lard, topical | … | … | … | … | 1 | 8.3 |
| **Total** | **40** | **100.0** | **60** | **100.0** | **12** | **100.0** |
