# Supplementary material for: Snakebites in “Invisible Populations”: A cross-sectional survey in riverine populations in the remote western Brazilian Amazon
Source: PLoS Negl Trop Dis. 2021 Sep 9;15(9):e0009758. doi: 10.1371/journal.pntd.0009758 (PMC8454940; doi:10.1371/journal.pntd.0009758)
Supplement: Attachment — Submitted filename: Dear Editor invisible snakebites.docx [file pntd.0009758.s005.docx]

Dear Editor,

Re: Manuscript **PNTD-D-21-00854** titled "Snakebites in “Invisible Populations”: A Cross-Sectional Survey in Riverine Populations in the Remote Western Brazilian Amazon"

We sincerely thank both reviewers for their considered, insightful and very helpful comments and suggestions, which we have used to improve the quality of our manuscript. We have also checked the manuscript meets journal formatting requirements.

Authors’ Responses to Reviewers:

Reviewer #1: The manuscript is well written and clearly shows the importance to know the magnitude of snakebite underreporting has to the healthy system constituting a serious problem and an aspect not addressed in a detailed form in the literature like this manuscript does it. 
The objectives are clear and the design appropriate, but I would like to know if the authors have the answers about the quantity of envenoming that occurs in the distinct seasons and if the floods river have some importance to this quantity. I think that this aspect was not mentioned and has some implications for the results obtained. In the discussion section, the authors mentioned something in an "en passant" manner. The methods are clear and appropriate for the study proposed.

RESPONSE: We thank the reviewer for recognizing the importance and relevance of this study for, eventually, helping to plan strategies to improve treatment access of snakebites in remote tropical settings.

We agree with the reviewer that information about the season in which snakebite occurred would be very relevant to understanding differences in access to healthcare. Unfortunately, this information was not added to the data collection instrument. We were unable to predict whether research participants would be able to recall this information, as many cases occurred many years ago.

We've added this limitation to the text, as follows (Lines 755-766):

*“The collection of information in this study depended on the memory of the interviewees, who in many cases were already elderly or had suffered snakebites that had occurred more than 10 years ago. Leveling and sharpening biases are also possible, with memory distortions introduced by the loss of details in a recollection over time. These are often concurrent with a selective recollection of certain details that take on exaggerated significance in relation to the details or aspects of the experience that are lost through leveling. For example, information about the season in which the snakebite occurred would be very relevant to understanding differences in access to healthcare. Unfortunately, such detailed information was not added to the data collection instrument, since research participants would be unable to recall this information, as a large number of cases occurred many years ago.”*

Reviewer #1: Results obtained are new and with an importance to the field. Snakebite underreporting is a real problem not only in Brazil but in other countries too. This aspect has many consequences to the health system that must be raised and debated for improvement.
It is important to mention or comment about the season of the year where the authors found the highest accidents, if it occurs in a specific season or not. Another aspect that can be addressed is the floods river and if this aspect impacts the numbers of accidents or not in these specific studied areas.

RESPONSE: We thank the reviewer for recognizing the importance and relevance of this study for, eventually, helping to plan strategies to improve treatment access of snakebites in remote tropical settings.

Regarding seasonality, please see previous response.

The authors conducted some health education during this trip with this invisible population?
RESPONSE: This is an excellent point that reviewer 1 raised. In our project, information on first aid in cases of snakebites, with an emphasis on the need to seek immediate medical care, as well as the nearest hospital to be accessed by participants, was provided by the field researcher.

We added this sentence to the manuscript (Lines 339-341):

*“After data collection, information on first aid in cases of snakebites, with an emphasis on the need to seek immediate medical care, as well as the nearest hospital to be accessed by participants, was provided by the field researcher.”*

Reviewer #1: The conclusions is supported by the results obtained with an importance to the field. Snakebite underreporting is a real problem not only in Brazil but in other countries too. This aspect has many consequences to the health system that must be raised and debated for improvement.
RESPONSE: We thank the reviewer for recognizing the importance and relevance of this study for, eventually, helping to plan strategies to improve treatment access of snakebites in remote tropical settings.

Reviewer #1: The manuscript must have to have page numbers as well as line numbers to the reviewers' scores are properly corrected.
RESPONSE: Added accordingly.

It is important to mention or comment about the season of the year where the authors found the highest accidents, if it occurs in a specific season or not. Another aspect that can be addressed is the floods river and if this aspect impacts the numbers of accidents or not in these specific studied areas. 
RESPONSE: Please see previous responses.

The authors conducted some health education during this trip with this invisible population?
RESPONSE: This is an excellent point that reviewer 1 raised. In our project, information on first aid in cases of snakebites, with an emphasis on the need to seek immediate medical care, as well as the nearest hospital to be accessed by participants, was provided by the field researcher.

We added this sentence to the manuscript (Lines 339-341):

*“After data collection, information on first aid in cases of snakebites, with an emphasis on the need to seek immediate medical care, as well as the nearest hospital to be accessed by participants, was provided by the field researcher.”*

Reviewer #2: Thank you for the opportunity to review this important cross-sectional study of snakebite in the Riverine populations of the Amazon. This is important research that gives us important information regarding under reporting of snakebite in this population. please consider the suggestions below:
RESPONSE: We thank the reviewer for recognizing the importance and relevance of this study for, eventually, improving the treatment of snakebite in remote tropical settings.

1. The manuscript is well written and the language flows well, but I do have a critique regarding its length. There are many areas of the manuscript that are not essential to the understanding of this specific study. I feel that some of the topics would be better served as publication a review article as opposed the this specific study. Examples include:
RESPONSE: Thanks for the suggestion. We removed a lot of text according the reviewer’s recommendation.

Methods Study sites. The first paragraph is certainly informative, but goes into a lot of detail that is important to snakebite in the Amazon generally, but not important to this specific study. Especially the part about the flooding, sedimentation, suspended solids, etc. Also the paragraph b beginning with "These communities present a model of land occupation..." Once again, this is important information but much to detailed to directly inform this study. I strongly suggest a separate review manuscript for this discussion. The length of the discussion is also too long for the reader to maintain focus on the primary study question. I would suggest removing almost all of the infectious disease part unless it informs snakebite. Only the parts to pertain directly the the under-reporting and the lack of access to healthcare should be retained for this manuscript. The conclusion can likewise be shortened.
RESPONSE: Thanks for the suggestion. We removed a lot of sentences according the reviewer’s recommendation.

2. Study design. This study is described as a quantitative study, yet there seems to be elements of a qualitative study as well. I cannot be certain from the methods described, but I would request clarity to determine if this is mixed methods or solely quantitative. An example of my uncertainty is the sentence "The inclusion of new participants finished once the saturation point had been reached." please describe "saturation". do you mean thematic saturation as is accepted in qualitative methods. If not is there another way the sample size was determined. Additionally the questionnaire had closed and open ended questions. Were the open ended questions used to determine thematic saturation. This can likely be easily addressed by giving a little more detail.
RESPONSE: In this new version of the manuscript, we clarified these points.

We preferred not to make a categorical classification into ‘quantitative’ or ‘qualitative’. By definition, the study was not carried out using a mixed method, as the basic premise of this methodology is that such integration of quantitative and qualitative data collection, using triangulation for example (Wisdom and Creswell, 2013). Otherwise, we present separately the data obtained in the two open questions on self-care procedures.

Regarding the process of saturation in the chain-referral (snowball) sampling technique (not thematic saturation actually), used in our study, saturation point was reached when the recruited participants no longer had snakebite cases in the community to indicate to the researcher for new interviews.

The following sentence was added to Methods (Lines 292-294):

*‘The inclusion of new participants finished once the saturation point had been reached, that is, when the recruited participants no longer had new snakebite cases in the community to indicate to the researcher.’*

Reference:

Wisdom J and Creswell JW. Mixed Methods: Integrating Quantitative and Qualitative Data Collection and Analysis While Studying Patient-Centered Medical Home Models. Rockville, MD: Agency for Healthcare Research and Quality. February 2013. AHRQ Publication No. 13-0028-EF.

3. As this article will be read by an international audience, I would like to point out some language that will be culturally troublesome for at least some the your readers. I strongly recommend that the word "Negro" be removed. This term is very offensive in the US. I also suggest removing the majority of the second paragraph that discusses genetics as it isn't vital to introduction or the study. Simply describe the population as admixed and remove the "genetic" part. To my knowledge we have no evidence that there is any important genetic difference between these various populations that impact snakebite outcomes. The current language unintentionally contributes to the paradigm of seeing these people as "other". Since this isn't a genetic study, I strongly suggest just removing.

RESPONSE: We thank the reviewer for these suggestions. The word ‘Negro’ was removed and ‘genetic’ part was summarized.
